# Optimal hepatobiliary scintigraphy for gallbladder dyskinesia^☆^

**DOI:** 10.1016/j.sopen.2020.10.003

**Published:** 2020-11-19

**Authors:** K.F. Flick, M. Soufi, C.M. Sublette, C.A. Sinsabaugh, CL. Colgate, M. Tann, M.G. House

**Affiliations:** a Department of Surgery, Indianapolis, IN; b Department of Radiology and Imaging Sciences, Indianapolis, IN; c Center for Outcomes Research in Surgery, Indianapolis, IN; d Indiana University School of Medicine, Indianapolis, IN

## Abstract

**Background:**

The accuracy of hepatobiliary scintigraphy to assess gallbladder function remains controversial. National supply shortages of pharmaceutical-grade cholecystokinin led to the use of an oral fatty meal to stimulate gallbladder contraction during hepatobiliary scintigraphy. The goal of this study was to compare the predictive indices of cholecystokinin and fatty meal ingestion for stimulation of gallbladder contraction.

**Methods:**

Patients evaluated with hepatobiliary iminodiacetic acid scan from 2014 to 2017 were reviewed and grouped based on testing stimulant (fatty meal versus cholecystokinin). Patients who later underwent cholecystectomy were selected for analysis. Hepatobiliary iminodiacetic acid results were correlated with surgical pathology and postoperative resolution of symptoms. Two-way statistical analysis was performed.

**Results:**

A total of 359 patients underwent hepatobiliary iminodiacetic acid scan followed by cholecystectomy for biliary dyskinesia. Patients who received fatty meal stimulant (n = 86) were compared to those that received cholecystokinin (n = 273). Mean gallbladder ejection fraction during hepatobiliary iminodiacetic acid was 38% and 44% for the cholecystokinin and fatty meal groups, respectively, P = .073. Predictive metrics were not statistically different between groups with regard to pathology, symptomatic improvement, or accuracy. Symptomatic resolution (cholecystokinin–hepatobiliary iminodiacetic acid 78%, fatty meal–hepatobiliary iminodiacetic acid 68%; P = 0.058) and specificity (cholecystokinin–hepatobiliary iminodiacetic acid 26%, fatty meal–hepatobiliary iminodiacetic acid 44%, P = 0.417) were comparable in both testing groups.

**Conclusion:**

Stimulation of gallbladder contraction with a fatty meal during hepatobiliary iminodiacetic acid testing is a more affordable and reliable alternative to cholecystokinin for patients undergoing evaluation for gallbladder dysmotility.

## Highlights

• Use of a fatty meal as a stimulant for hepatobiliary iminodiacetic acid scan.
• Affordable alternative to cholecystokinin for a hepatobiliary iminodiacetic acid scan.
• Comparison of 2 stimulants used for hepatobiliary iminodiacetic acid scan.
• Reliable alternative to IV cholecystokinin for hepatobiliary iminodiacetic acid scans.

## INTRODUCTION

Gallbladder dyskinesia is a disease that arises from uncoordinated function of the gallbladder, cystic duct, and sphincter of Oddi [1]. This functional disorder can result in gallbladder spasm, hyperviscosity of bile, and chemical cholecystitis even in the absence of stones. Whipple first recognized biliary dyskinesia in 1922 while evaluating patients suffering from biliary colic in the absence of a structural abnormality during orally provoked cholecystogram. This original article reported resolution of symptoms after cholecystectomy in 76% of patients [2]. In the current era, patients with biliary colic are typically evaluated with transabdominal ultrasound and serologic liver function testing (LFT). In the absence of cholelithiasis or LFT abnormalities, clinical providers may recommend further evaluation with cross-sectional imaging or a hepatobiliary iminodiacetic acid (HIDA) scan. Compared to other imaging modalities, HIDA scan can assess gallbladder function objectively and plays a major role in the evaluation of patients suspected to have gallbladder dyskinesia.

The etiology of gallbladder dyskinesia remains unclear, with a poor understanding of the pathophysiology that underlies the sequence of events involving gallbladder and biliary dysmotility, changes in bile composition, cholestasis, bile hyperviscosity, and chemically induced inflammation leading to chronic cholecystitis [3]. Similar to other visceral motility disorders such as delayed gastric emptying and colonic inertia, organ dysmotility is considered an essential component of gallbladder dyskinesia resulting in biliary pain. Although unreliable, reproducibility of symptoms during a HIDA scan is another factor associated with a diagnosis of gallbladder dyskinesia [[4], [5], [6]]. No gold standard for the diagnosis of gallbladder dyskinesia exists; however, HIDA scan is the test of choice to evaluate this disorder.

Gallbladder dyskinesia may also take the form of hyperkinesia. This functional disorder is also not fully understood but has been attributed to an increase in the concentration of CCK receptors or hypersensitivity of CCK receptors within the gallbladder that leads to vigorous contractile forces resulting in luminal hypertension causing mucosal injury, inflammation, and cholecystitis. Whereas the lower threshold of gallbladder ejection fraction (GBEF) with CCK during HIDA scan is established at 35%, the upper limit used to define hyperkinesia is quite variable, with an estimated range of 65–90% [[7], [8], [9]].

During a national supply shortage of CCK pharmaceutical analogue in 2014, many North American institutions were forced to transition to an oral stimulant to assess GBEF during HIDA scans for the evaluation of gallbladder dyskinesia. The purpose of this present study is to compare the performance of CCK and fatty meal (FM) ingestion on gallbladder contraction during HIDA scan for patients undergoing evaluation for gallbladder dyskinesia and to assess predictability for symptom resolution after cholecystectomy.

## METHODS

### Patient Cohort

The Indiana Network for Patient Care (INPC) collects clinical information from most hospitals within central Indiana. This system provides a community-wide clinical repository for accessing medical records for the purposes of health services research in compliance with institutional review board rules and regulations. All HIDA scans performed between January 2014 and December 2017 were accessed from the INPC. All participant hospitals used the same standardized protocol with no variability statewide. All hospitals moved to the FM substitute, Ensure, when a national shortage of CCK drug analogues halted the supply chain of Kinevac for use in HIDA imaging.

Indications for each nuclear medicine scan as well as GBEF measurements were collected. The entire patient cohort was cross-referenced to individual pathology reports containing gallbladder specimens, patient characteristics, and clinical information from the electronic medical record. Postoperative follow-up appointments were reviewed to identify whether surgery resulted in symptom resolution. Patients were categorized into 2 groups based on the provocative agent used during HIDA scan: oral FM or intravenous CCK.

Exclusion criteria included patients undergoing HIDA scan for the following listed indications: acute cholecystitis, cholelithiasis, bile leak, and functional evaluation after liver transplant, as well as those with reports lacking GBEF data.

### Imaging Protocol and Gallbladder Ejection Fraction Interpretation

CCK (sincalide) cholescintigraphy was performed according to the consensus recommendations of an interdisciplinary panel [5]. The radiopharmaceutical used for the test was Tc-99m-trimethylbromo-iminodiacetic acid [10]. This was administered at a dose of 6 mCi (222 MBq) via standard intravenous administration. One of two secretagogues was used for gallbladder stimulation:

1. CCK-HIDA: intravenous CCK analogue (Kinevac) was infused at a rate of 0.02 *μg*/kg/min for up to 60 minutes, and then anterior dynamic images were taken throughout the examination period.
2. 8 oz of anFM: The protocol for HIDA scanning with FM stimulation was standardized across the state. Patients begin drinking the FM, Ensure plus 240 mL, 60 minutes before the start of imaging. All patients must complete the meal to proceed with the test; otherwise, the test is canceled and rescheduled. Anterior dynamic images were taken 60 minutes after consumption.

Histogram analysis was used to determine the mean and standard deviation of each stimulant, FM versus CCK. A GBEF value less than 35% (CCK) or 38% (FM) was considered abnormal in these patients and was designated as hypokinesia based on accepted thresholds within the medical literature [[11], [12], [13]], whereas GBEF values greater than 80% (CCK and FM) were considered abnormal and a marker of biliary hyperkinesia [14].

### Statistical Analysis

Gallbladder ejection fractions from CCK and FM groups were compared with 2 outcomes after cholecystectomy: final pathology result and postoperative clinical information (resolution of symptoms and presence of any complication). *χ^2^* and Fisher exact tests were used to compare performance of CCK and FM stimulant in terms of predictive metrics. Statistical analyses were performed with R version 3.5.2 (Vienna, Austria).

## RESULTS

### Gallbladder Ejection Fraction for Each Stimulant

A total of 2,560 patients (1,175 FM vs 1,385 CCK) from hospitals within the state of Indiana were evaluated with HIDA scans for gallbladder disease. The mean [SD] GBEF reported for these scans that used an FM supplement was 67% [first standard deviation, SD, 22%]. Ten percent of these scans reported a gallbladder ejection fraction (< 35%) that qualified as hypokinesia. Concurrent analysis of CCK-stimulated HIDA scans demonstrated a mean [SD] GBEF of 55%, and 28% of patients were considered to have gallbladder hypokinesia. The mean GBEFs for the 2 stimulant groups were significantly different (*P* < .001). When CCK is used, HIDA scanning is more than 2.5 times more likely to report gallbladder hypokinesia with an ejection fraction of < 35%.

### Comparison of GBEF and Operative Volume Associated With Each Stimulant

**Fig 1:**
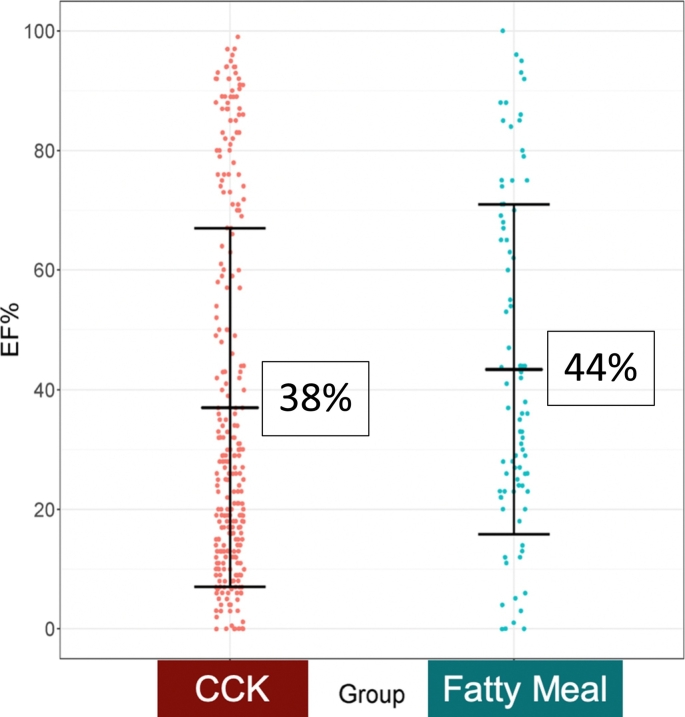
Gallbladder EF% distribution with mean and 1 standard deviation represented. A logistic regression analysis was performed which indicated no significant difference in mean [SD] GBEF between CCK (38% [27]) and Ensure (44% [27]) after adjusting for age, gender, race, and diabetes, *P* = .073. Abbreviations: *EF*, ejection fraction; *SD*, standard deviation.

All patients who underwent HIDA scan testing from 2014 to 2017 were reviewed. A total of 451 went on to undergo cholecystectomy. Ninety-two were excluded secondary to a diagnosis of acute cholecystitis or cholelithiasis, and 359 patients were included for final analysis. A total of 274 (76%) of these followed a preoperative CCK-provoked HIDA scan. The mean [SD] GBEF on HIDA scan testing was comparable for the 2 stimuli: CCK 38 [31]% and FM 44 [27]% (*P* = .073), Figure. 1. Despite this finding, the frequency of cholecystectomy correlated with stimulant type used for HIDA scintigraphy. The 2 groups based on stimulant exhibited similar demographics at baseline (Table 1).

**Table 1:** Baseline group comparisons

*Variable*	*Fatty meal* N *(%) or* N *[SD]*	*CCK* N *(%) or* N *[SD]*	P *value*
Number of patients	86	273	
Age (years), mean [SD]	38.2 [16.4]	43.0 [18.3]	0.1
Male sex	14 (16)	74 (27)	0.094
Race (white)	82 (95)	262 (96)	0.6
Diabetes mellitus	6 (7)	38 (14)	0.2
BMI (kg/m^2)^	30 [6.7]	31.3 [8.5]	0.2
Hypertension	24 (28)	82 (30)	0.7
Cirrhosis/NASH	16 (19)	71 (26)	0.2
COPD	4 (5)	19 (7)	0.5
Smoking history	19 (22)	57 (21)	0.9
CAD	8 (9)	27 (10)	1.0

*BMI*, body mass index; *CAD*, coronary artery disease; *CCK*, cholecystokinin; *COPD*, *Chronic Obstructive Pulmonary Disease*; *kg*, kilogram; *m*, meter; *N*, number; *NASH*, nonalcoholic steatohepatitis; *SD*, standard deviation.

The rate of cholecystectomy after a CCK-provoked scan was more than 2½ times the rate of operation after an FM-provoked scan. Specific postoperative outcomes included final surgical histopathology and improvement of symptoms after cholecystectomy. Comparison of outcome metrics between the 2 stimulants revealed no statistical differences in sensitivity or specificity for symptomatic improvement (CCK-HIDA 78%/26%, FM-HIDA 68%/44%; *P* = .058/*P* = .417) after cholecystectomy. Similarly, the type of stimulant used for HIDA scintigraphy was not associated with final pathology (CCK-HIDA 77%/12%, FM-HIDA 67%/43%, *P* = .066/*P* = .142) (Table 2). Overall accuracy concerning final pathology and symptomatic improvement did not show statistically significant differences (pathology *P* = .132; symptom improvement, *P* = .259).

**Table 2:** Predictive metrics of fatty meal versus*.* CCK

*Secretagogue*							*95% CI*
**Fatty meal**			**Pathology**		**Accuracy:**	0.65	
*EF 39–80%*		**Abnormal**	**Normal**	**Total**	**Sensitivity:**	0.67	[0.55–0.77]
	**Normal EF**	26 (30%)	3 (3%)	29 (33%)	**Specificity:**	0.43	[0.11–0.80]
	**Abnormal EF**	53 (62%)	4 (5%)	57 (67%)	**PPV:**	0.93	[0.82–0.97]
	**Total**	79 (92%)	7 (8%)	**86**	**NPV:**	0.10	[0.02–0.18]

**CCK**			**Pathology**				
*EF 35–80%*		**Abnormal**	**Normal**	**Total**	**Accuracy:**	0.74	
	**Normal EF**	58 (23%)	2 (1%)	60 (22%)	**Sensitivity:**	0.77	[0.72–0.82]
	**Abnormal EF**	198 (73%)	14 (5%)	212 (78%)	**Specificity:**	0.12	[0.02–0.40]
	**Total**	256 (94%)	16 (6%)	**272**	**PPV:**	0.93	[0.89–0.96]
					**NPV:**	0.03	[0.01–0.13]

**Fatty meal**		**Symptomatic improvement (outcome)**			**Accuracy:**	0.65	
*EF 39–80%*		**Yes**	**No**	**Total**	**Sensitivity:**	0.68	[0.56–0.78]
	**Normal EF**	24 (30%)	4 (5%)	30 (35%)	**Specificity:**	0.44	[0.14–0.79]
	**Abnormal EF**	50 (59%)	5 (6%)	55 (65%)	**PPV:**	0.91	[0.80–0.97]
	**Total**	76 (89%)	9 (11%)	**83**	**NPV:**	0.14	[0.04–0.33]

**CCK**		**Symptomatic improvement (outcome)**			**Accuracy:**	0.72	
*EF 35–80%*		**Yes**	**No**	**Total**	**Sensitivity:**	0.78	[0.72–0.83]
	**Normal EF**	48 (19%)	9 (4%)	57 (22%)	**Specificity:**	0.26	[0.15–0.45]
	**Abnormal EF**	175 (68%)	25 (10%)	200 (78%)	**PPV:**	0.88	[0.82–0.92]
	**Total**	223 (87%)	34 (14%)	**257**	**NPV:**	0.16	[0.08–0.28]

Abbreviations: *CCK*, cholecystokinin analogue; *CI*, confidence interval; *EF*, ejection fraction; *PPV*, positive predictive value; *n/a*, not available; *NPV*, negative predictive value.

*One patient was excluded from CCK (pathology) and FM (symptomatic improvement), whereas 16 were excluded from CCK (symptomatic improvement) because information was not available.

## DISCUSSION

Biliary dyskinesia accounts for nearly 20% of all cholecystectomies in adults and up to 50% in pediatric patient populations [15]. The rate of cholecystectomy for biliary dyskinesia in the United States exceeds that in Europe and the rest of the world by an estimate of 2- to 5-fold [16,17]. This regional disparity may reflect the low diagnostic accuracy of HIDA scintigraphy for gallbladder and biliary dyskinesia; however, past studies suggest that CCK-provoked HIDA scintigraphy can predict therapeutic benefit after cholecystectomy for patients with an abnormal GBEF [18,19]. In a large retrospective observational study involving 374 patients, more than 90% of the patients with a low GBEF experienced resolution of symptoms after undergoing cholecystectomy. The nuclear medicine testing protocol applied in that study used a rapid CCK-8 (sincalide) infusion at a rate of 0.02 *μg*/kg/min over a 3-minute period. As a result of the fast infusion rate, 69% of the healthy controls exhibited a GBEF of less than 35% [17]. Another study by Yap et al performed a prospective randomized controlled trial for patients with an abnormal GBEF on HIDA scintigraphy. This protocol utilized a 45-minute CCK infusion period followed by a calculation of GBEF at 60 minutes. Based on GBEF values collected from 40 healthy volunteers, a cutoff value of 40% was considered abnormal. All patients with a GBEF < 40% were randomized to either surgical or nonsurgical management. In the surgical treatment arm, more than 90% of patients experienced resolution of symptoms after cholecystectomy. The majority patients randomized to the nonsurgical arm continued to experience pain and ultimately required cholecystectomy to achieve satisfactory results [13].

These 2 studies established CCK-provoked HIDA scintigraphy as a reliable diagnostic test for biliary dyskinesia and as a predictive tool for resolution of biliary colic symptoms after cholecystectomy. In the present study, when GBEF was below 35% on a CCK-provoked HIDA scan, chronic cholecystitis was reported on final histopathology in 93% of cases. Comparable results (93% PPV) occurred with FM-HIDA testing. Although the details of the CCK infusion protocol and imaging interval have changed over time, the fundamental content of the diagnostic testing process has remained unchanged.

Despite differences in study protocols with regard to stimulation of gallbladder contraction, patients with abnormal ejection fraction calculations on HIDA scintigraphy experience therapeutic benefit from cholecystectomy [20]. Thus, a cheaper, more practical and widely available oral stimulant should produce equivalent results compared to pharmaceutical grade intravenous stimulants. Regardless of the effects of various stimulants to produce gallbladder ejection, clinical decision making relies on pretest probability of a diagnostic study and anticipated outcomes after definitive treatment [19,21].

In the present study, patients suspected clinically as having gallbladder disease were evaluated with HIDA scans and differentiated based upon the stimulant given at the time of the test. For all patients, including those who did not go onto surgery, mean GBEF after oral FM was significantly higher. However, when evaluating only patients who underwent surgery, GBEF was statistically similar between the 2 stimulants. Most importantly, the 2 stimulants produced statistically similar predictive metrics concerning symptomatic improvement and final pathology. Thus, FM stimulant may be an equivalent, much more affordable alternative.

Because of the relative unavailability and high costs of CCK (sincalide) cholescintigraphy, various FMs have been used in exchange as a physiological stimulus for this diagnostic test. At least 1 study has been performed comparing FM supplement cholescintigraphy to intravenous CCK directly. This study examined 13 healthy individuals with both methodologies but used a weight-based dose of fatty acids. Results showed that the FM stimulant had a wider range of GBEF values, which led to the conclusion that a percentile rather than a percent value should be used in the assessment [8]. A few years later, Bartel et al described a protocol using corn oil emulsion as an alternative to intravenous CCK analogue in a 30-patient sample. GBEF was calculated up to 90 minutes after the consumption, but this study and the one by Ziessman et al concluded that a 60-minute cutoff best segregates diseased from healthy patients [9,22]. Mean [SD] GBEF at 60 minutes was 47% [38] and similar to the present study's findings of 44% [27]. Contrary to this study, Ziessman et al used a lower limit of < 33% to indicate hypokinesia. Later, in 2015, Jain et al assessed a customized FM replacement in 61 symptomatic patients and 59 asymptomatic volunteers and found that 30 minutes was the appropriate time to assess GBEF [23]. Present studies demonstrate a great amount of variability in GBEF with various FM substitutes. Most of this is dependent on the amount of fat present in the substrate and the testing method [24]. Further studies should be performed to develop a standardized diagnostic protocol based on fatty stimulant used.

Studies estimate the false-positive rates of this test to be 20% [25], whereas the present study revealed a higher false-negative rate. This investigation included patients who had cholecystectomy despite normal findings of GBEF on nuclear imaging. Normokinetic GB dysfunction was hypothesized as the indication for operation in this patient subgroup and defined the false-negative group of patients. Outcomes after cholecystectomy for the false-negative group were compared to the group with an abnormal CCK-HIDA scan. Postcholecystectomy outcomes between the 2 groups did not differ. Final histopathology of gallbladder specimens demonstrated chronic cholecystitis in more than 90% of all patients. It is possible that surgical pathology may overdiagnose chronic cholecystitis or support an underlying disease process in the absence of radiologic findings [26].

Resolution of biliary colic symptoms was observed after cholecystectomy in 79% and 89% of the patients who underwent preoperative testing with CCK-HIDA and FM-HIDA, respectively. When cholecystectomy was performed in the setting of a normal GBEF on HIDA scan, 15% of patients did not experience resolution of symptoms. The presence of typical gallbladder dyskinesia symptoms appears to be a better predictor of postsurgical relief than an ejection fraction value according to 2 separate studies. Carr et al reported resolution of symptoms postcholecystectomy in 80% of patients with a normal GBEF on preoperative testing [27], whereas 1 meta-analysis including nearly 1,000 patients pooled from 9 studies discovered a positive outcome in more than 84% of individuals with a normal GBEF [27]. The retrospective nature of all published series limits the validity of these findings despite the apparent benefits of cholecystectomy irrespective of the findings of preoperative testing.

Clinical judgment seems to be the optimal modality for an accurate diagnosis of biliary dyskinesia that responds to cholecystectomy. And HIDA scan carries low yield when used outside of the context of clinical findings and symptoms consistent with gallbladder or biliary dyskinesia. However, this test is still ordered frequently in the state of Indiana and nationwide. Normal gallbladder ejection calculation during HIDA scan typically avoids cholecystectomy in a patient suspected of having gallbladder dyskinesia without cholelithiasis. Many clinical providers believe that CCK is the best stimulus for gallbladder contraction but fail to realize the cost associated with CCK drug analogues and their nonphysiologic effects. During a national shortage in the supply chain for CCK, testing centers were forced to use FM stimulation of endogenous CCK as a replacement. The shifting has result in significant saving in cost. Unfortunately, it is not feasible to acquire the exact cost for HIDA scanning at all testing centers included in this study. Among the highest volume testing centers, the average cost of a HIDA scan with calculation of gallbladder ejection fraction after CCK injection is US $1283, with US $388 allocated to the cost of the CCK drug analogue Kinevac.

Early referral for surgical evaluation may mitigate unnecessary expensive testing for the majority of patients suspected clinically to have a diagnosis of biliary dyskinesia. The Rome 4 criteria were designed to help objectify any significant clinical findings such as pain located in the right upper quadrant or epigastric region that lasts for a minimum of 30 minutes, is experienced intermittently for more than 3 months, and is not associated with bowel movements more than 20% of the time. Furthermore, the pain must be severe enough to interfere with daily activities or result in an emergency room visit. Other characterizations of pain such as associated nausea, vomiting, radiation to the back, and waking up from sleep are only supportive and not essential for the diagnosis. An abnormal GBEF on scintigraphy for a patient with an acalculous gallbladder and normal serum liver function test results is supportive but not confirmatory for a diagnosis of biliary dyskinesia [19,26,28,29]. A physiologic stimulant such as FM should always be considered with few exceptions. Biliary dyskinesia is a common issue after gastric bypass operations that results in many alterations in gut physiology including decreased stimulation of gallbladder contraction. Similarly, any operation that results in duodenal bypass will reduce the reliability of an FM stimulant for gallbladder contraction. Other situations that would favor a CCK analogue over fatty meal stimulation include gastroparesis and exocrine pancreatic insufficiency when high CCK levels are often noted because of low proteolytic enzymes.

Interesting differences were observed for patients with abnormal GBEF on FM- or CCK-HIDA testing. Patients who were evaluated with CCK-HIDA were far more likely to have an abnormal GBEF finding. Thus, patients were more likely to undergo cholecystectomy after CCK-HIDA compared to FM-HIDA. The retrospective nature of this study does not permit an opportunity to study how HIDA findings influence surgical decision making for patients with a suspected diagnosis of BD. Furthermore, unblinded biases affect the reporting of symptom resolution in the postoperative period after cholecystectomy.

## Author Contributions

Flick, KF: study design, data acquisition, data analysis, manuscript writing /editing. Soufi, M: study design, data acquisition, data analysis, manuscript writing /editing. Sublette, CM: data acquisition, data analysis. Sinsabaugh, C: data acquisition, data analysis. Colgate, CL: data analysis, statistics. Tann, M: study design, manuscript writing/editing. House MG: study design, data analysis, manuscript writing /editing.

## Conflict of Interest

None declared.

## Funding Sources

No external funding or support was provided for this study.

## Author Contribution

Katelyn F Flick: Conception, data curation, investigation, methodology, original drafting, review and editing of manuscript.

Christopher M Sublette: Conception, data curation, original drafting, review and editing.

Mazhar Soufi: Conception, data curation, investigation, methodology, original drafting, review and editing of manuscript.

Cameron L Colgate: Formal analysis, methodology, review and editing of manuscript.

Christopher A Sinsabaugh: Conception, data curation, investigation, methodology, original drafting, review and editing of manuscript.

Mark Tann: Conception, data curation, investigation, supervision, validation, review and editing of manuscript.

Michael G. House: Conception, investigation, methodology, supervision, validation, review and editing of manuscript.
